# Effect of Conventional and Cathodic Cage Plasma Nitriding on the Mechanical and Corrosion Behavior of AISI 420 Martensitic Stainless Steel

**DOI:** 10.1021/acsomega.5c08647

**Published:** 2026-01-13

**Authors:** André Felipe Soares do Monte e Silva, Maxwell Santana Libório, Luciano Lucas Fernandes Lima, Thércio Henrique de Carvalho Costa, Michelle Cequeira Feitor, Juliermes Carvalho Pereira, Renan Matos Monção, João Rodrigues de Barros Neto, Ediones Maciel de Sousa, Anthunes Íkaro de Araújo, Rômulo Ribeiro Magalhães de Sousa

**Affiliations:** † Post-Graduate Materials Science and Engineering Program, 67823 Federal University of Piauí, Teresina, PI 64058-074, Brazil; ‡ School of Science and Technology, Federal University of Rio Grande do Norte, Natal, RN 59078-900, Brazil; § Department of Materials, Federal University of Rio Grande do Norte, Natal, RN 59078-900, Brazil; ∥ Postgraduate Program in Mechanical Engineering, Federal University of Rio Grande do Norte, Natal, RN 59078-900, Brazil; ⊥ Department of Mathematics and Physics, State University of Maranhão, Caxias, MA 65600-120, Brazil; # Materials Engineering Department, Federal University of Piauí, Teresina, PI 64002-150, Brazil; ∇ Department of Mechanical Engineering, Federal Institute of Education, Science and Technology of Maranhão, São Luís, MA 65073-000, Brazil; ○ Postgraduate Program in Materials Science and Engineering, Federal University of Rio Grande do Norte, Natal, RN 59078-900, Brazil; ◆ Department of Mechanical Engineering, Federal University of Piauí, Teresina, PI 64049-550, Brazil

## Abstract

AISI 420 martensitic stainless steel was treated via conventional plasma nitriding (PN) and cathodic cage plasma nitriding (CCPN) at temperatures ranging from 400 to 500 °C for 5 h to enhance surface properties. X-ray diffraction and scanning electron microscopy analyses confirmed uniform nitride layer formation, and microhardness measurements showed a peak hardness of 1270 HV_0_._5_ for PN at 500 °C. Notably, CCPN at 450 °C achieved similar hardness (1011 HV_0_._5_) alongside excellent adhesion (HF1–HF2), highlighting the effectiveness of the cathodic cage technique. Electrochemical impedance spectroscopy and open-circuit potential tests in 3.5 wt % NaCl showed improved corrosion resistance in all nitrided samples compared to the untreated steel. These results indicate that specific nitriding conditions, particularly PN at 450 °C and CCPN at 400 °C, achieve an optimal balance of hardness, corrosion protection, and interfacial toughness while preserving the substrate’s inherent microstructure.

## Graphical abstract

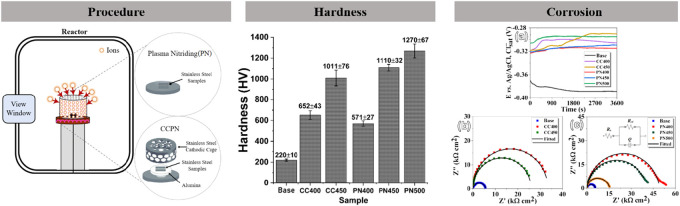

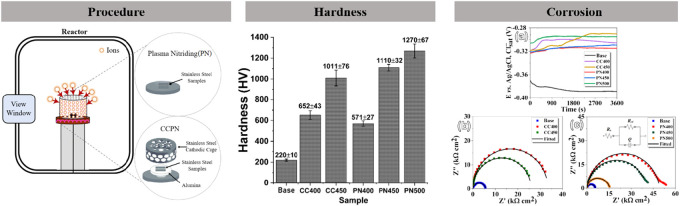

## 1 Introduction

AISI 420 martensitic stainless steel (420 MSS) is widely recognized for its outstanding mechanical properties, including high hardness and moderate corrosion resistance. This material is widely used in applications where these attributes are essential, such as the manufacture of tools, automotive parts, surgical instruments, and various industrial components.^1–3^ The composition of 420 MSS, which typically includes around 13% chromium and 0.2% carbon, provides it with a robust martensitic structure suitable for mechanically demanding environments.^4^ The combination of mechanical strength and corrosion resistance makes 420 MSS a preferred material for applications that demand durability and reliability under demanding service conditions.

Despite its favorable properties, AISI 420 stainless steel has some limitations. Its original hardness (220 HV_0_._5_) may fall short of requirements for high resistance to indentation and plastic deformation under concentrated loads.^5,6^ Likewise, its corrosion resistance in aggressive environments is only fair, leaving it prone to localized pitting and crevice attack over time.^2,7^ These shortcomings underscore the need to enhance both the surface hardness and corrosion resistance to extend the lifespan and reliability of 420 MSS components.^8^

Surface engineering techniques represent an effective strategy to overcome the limitations of AISI 420 stainless steel. By modification of the surface characteristics, it is possible to significantly enhance surface hardness and corrosion resistance, thereby improving the overall performance of the material. Several advanced surface engineering methods have been developed and extensively studied, including chemical vapor deposition (CVD)^9^, physical vapor deposition (PVD)^9^, laser nitriding^10,11^ , and plasma nitriding.^3,12,13^ Each of these techniques has unique advantages and applications, contributing to the optimization of surface properties in different contexts.

Among these techniques, plasma nitriding stands out as a particularly effective method for improving the surface properties of stainless steels. Plasma nitriding involves the diffusion of nitrogen into the steel’s surface, forming a hard nitrided layer.^3^ This process can significantly enhance the surface hardness and tribological properties of the material without compromising its core mechanical properties. Plasma nitriding is recognized for its ability to produce a nitrided layer with exceptional adhesion, making it a preferred choice for industrial applications where durability and hardness are essential.^14,15^

However, one of the main limitations of the conventional plasma nitriding process is the potential for chromium nitride precipitation, which can reduce the free chromium content in the solid solution and consequently diminish the corrosion resistance of the treated material.^16,17^ This issue is particularly pronounced at higher nitriding temperatures, where the formation of chromium nitrides becomes more prominent.^18^ To address these limitations, the plasma nitriding with a cathodic cage (CCPN) technique has been employed. This method involves using a metallic cage, made of the same elements as the sample, connected to the cathodic potential. In this configuration, the material from the cage is eroded by the plasma and deposited onto the sample surface, promoting a controlled surface modification. This approach has shown promising results, particularly by enhancing the uniformity of the treatment and providing greater control over deposition parameters, resulting in more homogeneous coatings with optimized properties.^19^

This study investigates the effects of conventional and cathodic cage plasma nitriding on the surface properties of AISI 420 martensitic stainless steel. By comparing these two techniques, we aim to determine the optimal nitriding parameters that achieve the best balance of surface hardness and corrosion resistance. This research contributes to the ongoing development of advanced surface engineering methods and provides information about the practical applications of CCPN in enhancing the performance of martensitic stainless steels.

## 2 Materials and Methods

In this study, samples of AISI 420 stainless steel, having the composition (wt %) 12.64 Cr, 0.22 C, 0.32 Mn, 0.33 Ni, 0.44 Si, 0.03 S, 0.03 P, and balance Fe, were used as substrates. The samples were prepared with a thickness of 2 mm and a diameter of 20 mm from a metallic sheet, and the steel’s initial hardness was measured at 220 HV_0_._5_. The sample preparation process followed the method reported in detail in previous works to remove irregularities, oxidation, and contaminated surface layers, facilitating uniform nitrogen absorption and achieving a more homogeneous distribution of the nitride layer formed.^20^ Then, the samples were dried with hot-air jets and forwarded to their respective treatments.

Plasma treatments were performed in a plasma reactor connected to a vacuum pump with a base pressure of 0.2 mbar and a DC source with a maximum voltage of 1200 V, as illustrated in Figure 1 and detailed in a previous work.^21–23^ Both plasma nitriding (PN) and cathodic cage plasma nitriding (CCPN) included a presputtering stage in the same plasma equipment using only inert gases (H_2_/Ar = 1) at a temperature of 350 °C and a pressure of 1.3 mbar for 1 h. This step was essential for removing residual impurities and creating void spaces on the sample surfaces, thereby facilitating nitrogen diffusion. Following the presputtering, the PN and CCPN processes were conducted for 5 h at various temperatures and atmospheric conditions, as shown in Table 1. The change in the N_2_/H_2_ mixture ratio between the PN and CCPD processes is due to the surface modification mechanism characteristic of each plasma treatment technique. In PN, the atmosphere must contain more H_2_ to minimize cathode contamination, whereas in CCPN, a higher N_2_ content is required to enhance the sputtering effect in the cathode cage. These mixing ratios are widely reported in the literature.^24–27^ For the CCPN process, the sample was placed on an alumina disk under a floating potential.

**Figure 1:**
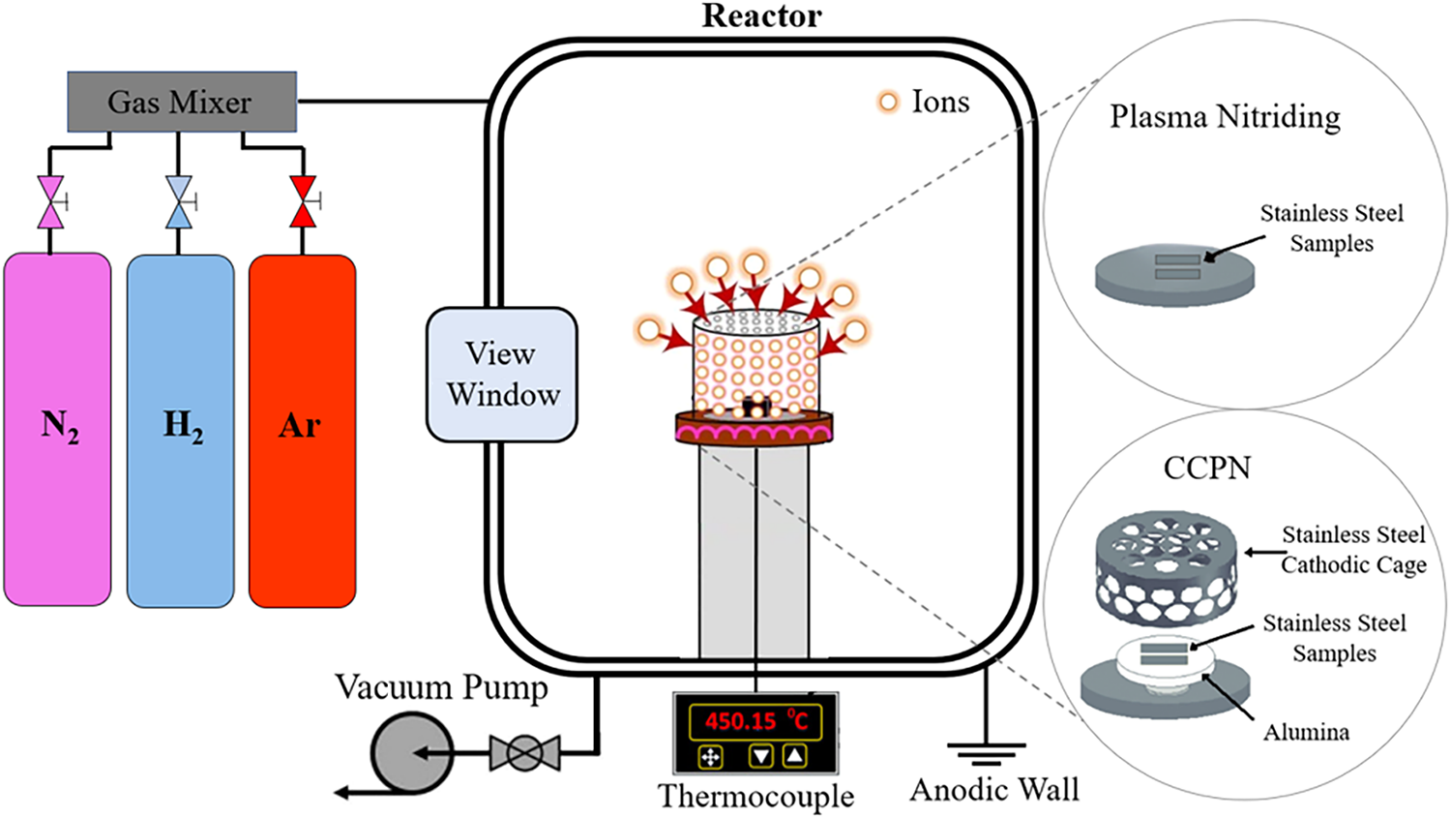
Schematic diagram of PN and CCPN highlighting the difference between the treatments.

**Table 1:** Processing Temperatures in PN and CCPN

sample	procedure	temperature	atmosphere
CC400	cathodic cage plasma nitriding (CCPN)	400 °C	75%N_2_–25%H_2_
CC450		450 °C	
PN400	plasma nitriding (PN)	400 °C	25%N_2_–75%H_2_
PN450		450 °C	
PN500		500 °C	

The crystalline phases of the coatings were determined by X-ray diffraction using a Shimadzu XRD-7000 instrument with Cu–Kα radiation. The copper target tube was subjected to 40 kV and 30 mA. A scanning range of 20–80° was used. Micrographs were obtained using a field emission scanning electron microscope (FE-SEM) (model: Quanta FEG 250 (FEI)), operating at accelerating voltages ranging from 1 to 30 kV. The system is equipped with an energy-dispersive X-ray spectroscopy (EDS) detector based on silicon drift detector (SDD) technology (model: Quantax EDS with XFlash 5010 (Bruker)). The acquisition parameters (beam energy, spot size, and magnification) are indicated at the bottom of each micrograph, along with the scale bar and magnification (notation: SE (secondary electron detector, ETD-SE)). Samples were mounted on aluminum stubs using a double-sided carbon adhesive tape and ground with carbon paint. Elemental microanalysis by EDS was performed at 20 kV with a spot size of 5.5. The Vickers microhardness (HV) test was performed with an Insize microhardness tester (model: ISH–TDV 1000 A-B). The load used was 50 gf applied for 15 s. Five measurements were taken to obtain the mean and standard deviation.^28^ A minimum spacing of 2.5 times the average length of the indentation diagonals was adopted in accordance with ASTM E-384.^29^ In addition, the edges of the samples were not analyzed due to the characteristic edge effect of nitriding.

Corrosion tests were performed with a three-electrode system in a glass cell using a 3.5% (m/m) NaCl solution (Dynamics 99%). An Ag/AgCl/KCl (sat.) electrode was used as a reference to obtain the potentiodynamic polarization curve. Potentiodynamic polarization measurements were initiated at −400 mV and terminated when the current reached 1 mA.cm^–2^ at a sweep rate of 1 mV.s^–1^. The open-circuit potential (OCP) was measured for 60 s before each potentiodynamic polarization measurement.

Corrosion tests were carried out in a conventional three-electrode electrochemical cell connected to a potentiostat/galvanostat. AISI 420 steel electrodes, either untreated or plasma nitride with an exposed area of 1.32 cm^2^, were used as working electrodes. A platinum plate (1 cm^2^) and an Ag(s)|AgCl(s)|Cl^–^ (sat. KCl aq.) wire served as the counter and reference electrodes, respectively. All electrodes were immersed in a 3.5 wt % NaCl solution at 23 °C, inside a Faraday cage to minimize electromagnetic interference. Initially, all electrodes remained immersed in the electrolyte for 1 h to monitor the open-circuit potential (OCP) until a steady state was reached. Electrochemical impedance spectroscopy (EIS) experiments were then conducted by applying a sinusoidal voltage perturbation with an amplitude of 10 mV over a frequency range from 6 mHz to 20 kHz. The electrochemical parameters were obtained by fitting the Nyquist plots.

The adhesion test was performed in accordance with ISO 6508–1, using an Insize Rockwell-C indenter (model: ISH-BRV) and a load of 150 kgf. The evaluation followed the Daimler-Benz scale (HF0–HF6) in accordance with BS ISO 26443:2016, where HF1–HF2 indicates excellent adhesion without significant delamination.

## 3 Results and Discussion

The SEM images in Figure 2 show the changes in surface morphology induced by conventional plasma treatment (panels (b), (d), and (f)) and cathodic cage plasma nitriding (panels (c) and (e)). As shown in Figure 2a, the untreated surface appears smooth with scratches resulting from the metallographic preparation process. The samples treated by the conventional method exhibit surface grain boundaries similar to those observed in the diffusion zone when nitrogen diffuses into the metallic matrix, whereas the samples treated with the cathodic cage show particle deposition due to the CCPN deposition effect. The morphology induced by the CCPN treatment resembles the composite nitride layers commonly reported for this technique in the literature.^30–32^ The EDS results (Table 2) show that the main surface elements on the treated samples are Fe, Cr, and N, indicating that nitrogen was present on the surfaces of all samples. As expected, all samples exhibited an increase in the nitrogen content with increasing treatment temperature. The samples subjected to conventional treatment, particularly PN400, do not exhibit a defined pattern in elemental weight percentages. The PN400 sample, in particular, exhibits a high iron content and moderate nitrogen concentrations. In contrast, in the PN450 and PN500 samples, the iron content is lower and the nitrogen levels are higher. This result can be attributed to optimal treatment conditions for the formation of iron nitrides, which favors their formation without promoting oxide formation.

**Figure 2:**
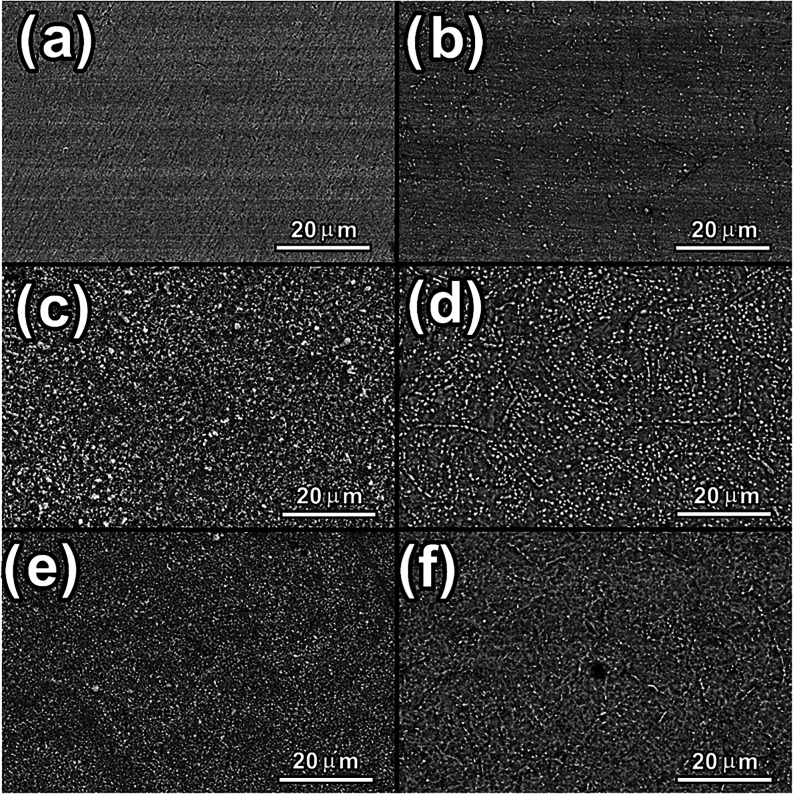
Surface SEM images of the samples: (a) substrate, (b) PN400, (c) CC400, (d) PN450, (e) CC450, and (f) PN500.

**Table 2:** Topographic EDS of Samples Treated by Conventional Nitriding and Cathodic Cage Nitriding and without Treatment

sample	Fe[at.%]	Cr[at.%]	N[at.%]
substrate	86.13	13.87	0.00
CC400	78.14	12.23	9.63
CC450	71.94	11.74	16.32
PN400	79.95	13.19	6.86
PN450	67.73	12.48	19.79
PN500	63.04	11.38	25.58

The phase compositions in the nitrided layer were analyzed with XRD by using Co–K_α_ radiation. As shown in Figure 3, XRD analysis of AISI 420 martensitic stainless steel samples subjected to plasma nitriding (PN) and cathodic cage plasma nitriding (CCPN) at various temperatures revealed significant differences in the phase composition. For the samples treated with PN at 400 °C (Figure 3b), the XRD patterns showed the presence of expanded martensite (α’N).

**Figure 3:**
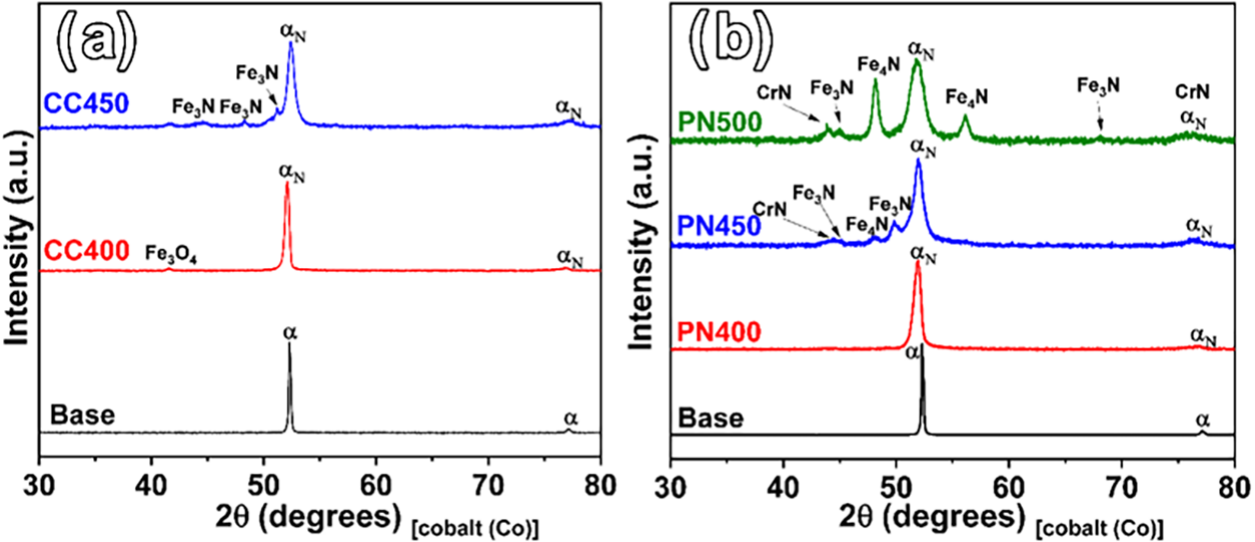
X-ray diffraction patterns of the samples untreated and treated at different temperatures: samples submitted to (a) CCPN and (b) PN.

Increasing the treatment temperature to 450 °C results in a decrease in the intensity of the α′N peaks, accompanied by the appearance and intensification of peaks attributed to nitride precipitates (Fe_3_N, Fe_4_N, and CrN). This pattern indicates that at this temperature, nitrogen saturation at the surface occurs, promoting nitride formation rather than incorporation into the expanded martensite structure. At 500 °C, the Fe_4_N phase becomes more pronounced, together with increased signals of Fe_3_N and CrN. It is important to note that the significant formation of these nitride precipitates is commonly associated with reduced corrosion resistance in treatments within this temperature range.^33^

In the samples treated with PN, an increase in the peak intensities of nitride phases (Fe_3_N, Fe_4_N, and CrN) is observed, indicating their formation and growth due to the treatment. The increase in surface temperature, combined with the application of the cathodic potential to the substrate, intensifies the diffusion of nitrogen into the crystal lattice. This enhanced diffusion introduces internal stresses, leading to a reduction in the material’s crystallinity, as evidenced by the broadening of the peaks in the diffractograms.^31^

Figure 3a shows that for plasma nitriding with a cathodic cage at 400 °C, the formation of nitrides did not occur in the material. However, nitrogen diffusion was also observed in the structure, evidenced by the displacement of the characteristic iron peak to smaller angles. At 450 °C, a layer of iron nitrides forms, and its formation is enhanced at higher temperatures. This is due to the principle of the CCPN technique, in which the plasma is generated in the cage rather than directly on the surface, leading to nitrides derived mainly from pulverized iron nitride particles from the cage, which are then deposited onto the surface.^32^

The surface microhardness results for the samples are listed in Figure 4. It is observed that the microhardness increases gradually on the surface as the treatment temperature rises, confirming the formation of a nitride layer with high hardness. The highest hardness value (1270 HV_0.5_) observed in the PN500-treated sample can be attributed not only to the presence of iron nitrides but also to the formation of hard CrN precipitates on the surface, as reported by Li et al. (2017).^32^

**Figure 4:**
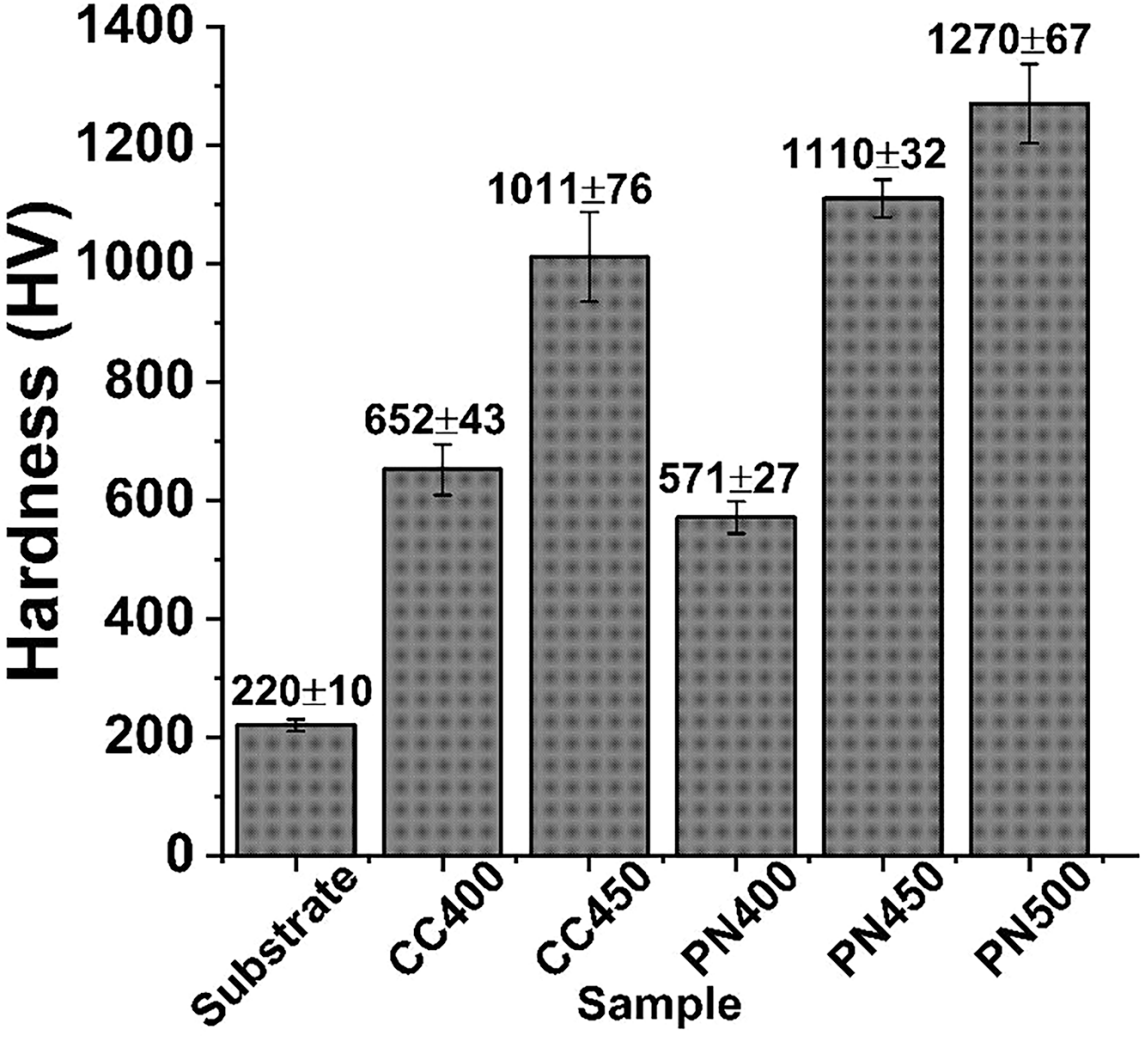
Surface microhardness of the untreated and treated samples.

All treated samples, both by the conventional method and the cathodic cage method, exhibited an increase in surface hardness, ranging from 2.73 times (PN400) to 5.77 times (PN500) higher than that of the untreated substrate. The samples treated with the cathodic cage method showed a slightly lower reduction in hardness than those treated with the conventional method, with values of 652 HV_0.5_ for CC400 and 1011 HV_0.5_ for CC450. This result also suggests the formation of a layer of nitride iron composites.

Figure 5 presents cross-sectional electron micrographs of the nitrided samples (panels (a–c) show samples treated by conventional plasma nitriding and panels (d) and (e) show samples treated using the cathodic cage technique). In the samples treated at 400 °C, no well-defined nitrided layer is observed; only the formation of expanded martensite is visible, attributed to nitrogen diffusion. At temperatures above 450 °C, a composite layer becomes evident in all samples, with thicknesses of approximately 40.94 μm (PN450), 76.74 μm (PN500), and 47.72 μm (CC450). The layer thickness increases with treatment temperature due to enhanced plasma kinetics at higher temperatures, and this region results from nitrogen diffusion followed by nitride formation.^34^ In addition, specific surface morphologies visible in the micrographs indicate the presence of an oxide, which is caused by the greater susceptibility of chemically etched surfaces. Despite differences in the layer composition, such as the formation of chromium nitrides in conventionally treated samples, as shown by XRD, all treated specimens exhibit similar layer thicknesses, indicating that the cathodic cage technique does not limit the development of comparable layers.^32^ These microstructural changes are restricted to the surface of the material and do not compromise the substrate’s inherent microstructure, ensuring that the core material retains its original properties while delivering a superior performance with minimal alteration.

**Figure 5:**
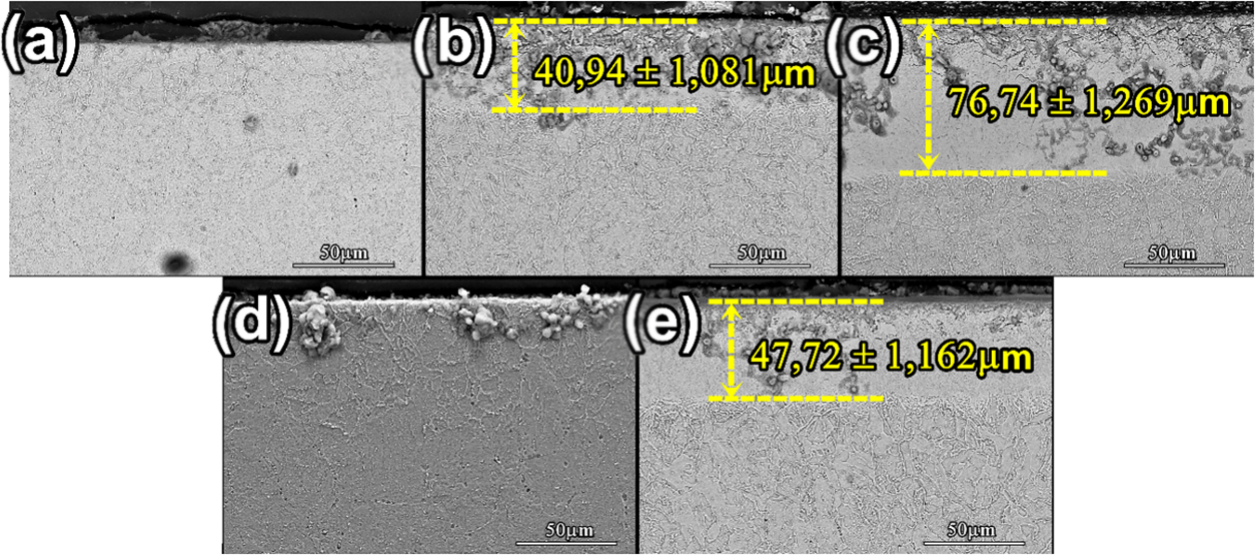
Transversal SEM images of samples treated by (a) PN400, (b) PN450, (c) PN500, (d) CC400, and (e) CC450.

Figure 6 presents the results obtained from the corrosion tests conducted in an electrolytic solution. Figure 6a shows the evolution of the open-circuit potential (OCP) over 1 h (3600 s) of testing. The untreated (substrate) sample exhibited the most negative potential values throughout the test, reaching even lower values over time (−388 mV at the end of the test). This behavior indicates increased electrochemical instability of the surface and, consequently, higher susceptibility to corrosive attack.^35^ In contrast, the plasma-treated samples showed higher noble OCP values within the evaluated time interval. Samples such as PN400, PN450, and PN500 exhibited a potential stability in the early stages of the test. Notably, the sample CC450 showed a continuous increase in the OCP over time, indicating progressive surface stabilization due to the formation of a passive layer and the iron nitride-based coating deposited during the CCPN process (see Figure 5).

**Figure 6:**
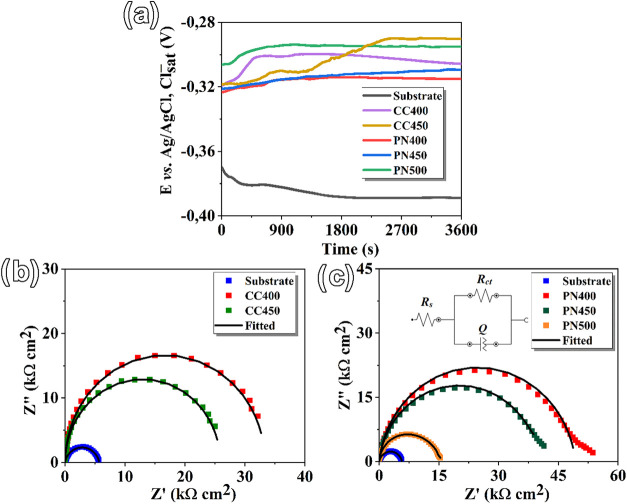
Corrosion test results: (a) open-circuit potential (OCP) monitoring; (b) Nyquist diagrams of samples nitrided using the cathodic cage technique; and (c) Nyquist diagrams of samples treated by conventional plasma nitriding.

Impedance spectra were used to evaluate the barrier effect of the coatings against substrate dissolution. The Nyquist plots of all coated samples exhibit high-frequency capacitive semicircles associated with interfacial charge transfer processes. The plots shown in Figure 6b,c present the real (*Z*′) and imaginary (−*Z*′) components of impedance for both the untreated AISI 420 sample (substrate) and the plasma-treated surfaces under different processing conditions.^36^ Semicircles with larger diameters, such as those observed for PN400, PN450, and CC400, are indicative of higher polarization resistance values.^37^

Table 3 presents the final OCP values obtained at the end of the corrosion test along with the constant-phase element *Q* parameters and resistance values extracted from the EIS data using a Randles equivalent circuit. In this circuit model, as shown in Figure 6b, *R*_S_ represents the solution resistance of the testing electrolyte between the working electrode and the reference electrode, while *R*_ct_ corresponds to the charge transfer resistance (associated with the reaction occurring between the substrate and the test solution). According to Azzi et al.,^35^ EIS modeling requires the use of a constant-phase element (Q), which often replaces the ideal capacitor since, in practice, the capacitive response is distorted by surface roughness, chemical heterogeneity, and coating irregularities. Although it is not a real capacitance, it is possible to calculate the effective capacitance value (*C*_eff_) from the Hsu–Mansfeld relation (eq 1)^38^, thus allowing an approximate quantification of the charge storage capacity at the interface.

1 $$C_{eff}\left(F\right)={\left[\frac{Y_{o}}{{\left(R_{s}^{-1}+R_{ct}^{-1}\right)}^{\left(1-n\right)}}\right]}^{1/n}$$

where *Y*_0_ and *n* are parameters of the *Q* element. The parameter *n* reflects the physical meaning of the *Q* element, such that *n* = 1 represents an ideal capacitor, *n* = −1 represents an ideal inductor, and *n* = 0 corresponds to a pure resistor.^39^

**Table 3:** Values of Solution Resistance (*R*_c_), Admittance (*Y*_o_), Exponent (*n*), Electrical Double-Layer Capacitance (*C*_dl_), and Charge Transfer Resistance (*R*_ct_) of the Samples Obtained from Fitting Experimental Data Using the Randles Circuit

sample	OCP/mV	*R*_s_ (Ω cm^2^)	*Y*_o_ (μMho s*^n^*)	*n*	*C*_dl_ (μF cm^–2^)	*R*_ct_ (kΩ cm^2^)
substrate	–388	5.6	1.31	0.87	0.53	5.728
CC400	–305	6.1	0.66	0.95	0.34	33.285
CC450	–290	7.5	1.68	0.91	0.55	25.840
PN400	–314	6.5	0.41	0.92	0.13	49.279
PN450	–309	4.12	0.62	0.92	0.20	39.572
PN500	–295	8.3	0.99	0.87	0.26	14.943

The *R*_*S*_ values range from 4.12 to 8.3  Ω.cm^2^, indicating that the electrolyte solution had good conductivity and was consistently standardized across all analyses. The *n* values presented in Table 3 suggest that all coatings exhibited a nonideal capacitive behavior, indicating good homogeneity and uniform ion diffusion within the coatings. This implies that the coating structures are uniform and that ion mobility at the interface does not encounter significant barriers or preferential pathways, such as large discontinuities.^40^ The effective capacitance (*C*_eff_), extracted from the parameters of the Q element, is proportional to the contact area between the working electrode and the electrolyte. This parameter shows that the substrate and CC450 samples have the highest contact area among all evaluated conditions (5.3 and 5.5 × 10^–7^ F/cm^2^). The behavior observed in the substrate results from the absence of any surface treatment, while in the CC450 sample, it may be attributed to the increased surface roughness introduced by the plasma modification.

The nonideal capacitive behavior, expressed by *n* < 1, also indicates that the sample surfaces have a low quantity and small size of pores.^35^ The *R*_ct_ values further show that the PN400 sample exhibits high resistance to ionic transfer within the pores, which is associated with smaller pore sizes, as can also be observed in the micrograph presented in Figure 2.

The electrochemical parameters obtained by fitting the EIS data to a Randles equivalent circuit, namely, solution resistance (*R*_s_), admittance (*Y*_o_), exponent (*n*), double-layer capacitance (*C*_dl_), and charge transfer resistance (*R*_ct_), are summarized in Table 3.

The results of the adhesion test are shown in Figure 7. These findings are consistent with the microhardness measurements, revealing brittle fracture and small cracks at the edges of the indentation in samples b–e, which correspond to those with the highest hardness values. Delamination of the nitrided layer near the edge of the indentation can also be observed in Figure 6e, confirming the presence of a hard laminar layer on the sample surface.^41–43^

**Figure 7:**
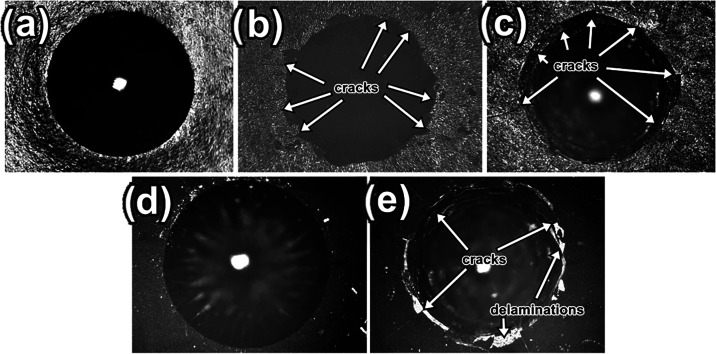
Adhesion test of the nitride layers of samples treated by conventional and cathodic cage nitriding. (a) PN400 HF1: no significant failures; (b) PN450 HF2: radial cracks, no spallation; (c) PN500 HF3: pronounced cracks, but no removals; (d) CC400 HF1: no significant failures; and (e) CC450 HF5: moderate delamination with localized removal.

The Rockwell-C adhesion test (150 kgf, ISO 6508–1/BS ISO 26443) revealed a clear dependence of the failure mode on the coating type and hardness. Plasma-nitrided samples showed progressively harsher crack patterns with an increasing treatment temperature: PN400 exhibited HF1 (no significant failures), PN450 exhibited HF2 (fine radial cracks without spallation), and PN500 exhibited HF3 (pronounced radial cracks but an intact film). In contrast, cathodic cage plasma nitriding (CCPN) maintained HF1 at CC400 but then suffered from HF5 at CC450 (moderate peripheral buckling with localized spallation). As the hardness increases, nitride coatings become more brittle and prone to crack initiation under the high localized stresses of the Rockwell indenter. SEM/EDS and XRD analyses confirmed the formation of FeN and CrN precipitates on the surface of the modified steels, which increased the hardness and reduced the toughness and adhesion. PN400 moderate hardness (∼652 HV_0_._5_) retains sufficient ductility to absorb indentation stresses, whereas the denser nitride networks in PN500 (∼1270 HV_0_._5_) act as crack propagation paths, leading to HF3. The deformation observed in the indentation on the CC450 sample indicates the presence of a hard, nitride surface film. However, unlike the adhesive behavior of the other samples’ coatings, this one showed the poorest adhesion to the substrate. In Figure 6e, it is possible to observe the formation of circumferential crack rings with several radial cracks initiated at the edge of the indentation. The contrast in the micrograph allows for the identification of delamination in some segments of the coating that extends beyond the edge of the indentation.^44^ Since the ring is not a continuous structure, the coating adhesion is classified as HF5 according to VDI 3198.^45^

## 4 Conclusions

In this work, it was demonstrated that both conventional plasma nitriding (PN) and cathodic cage plasma nitriding (CCPN) effectively enhance the surface performance of AISI 420 martensitic stainless steel by promoting the formation of hard, nitrided layers without compromising the substrate’s inherent microstructure. The PN process at 500 °C yielded the highest surface hardness (1270 HV_0_._5_) due to the abundant formation of iron and chromium nitrides, while CCPN treatments also produced substantial hardness increases (652 HV_0_._5_ at 400 °C and 1011 HV_0_._5_ at 450 °C), along with favorable corrosion resistance. Electrochemical impedance spectroscopy and open-circuit potential measurements showed that all plasma-treated samples exhibited a more noble and stable corrosion behavior in 3.5 wt % NaCl, with PN400 presenting the highest polarization resistance and CC450 displaying a continuously increasing OCP, indicative of robust passive layer formation.

However, the increased hardness observed at higher temperatures came at the expense of coating toughness and adhesion; PN500 samples showed pronounced radial cracking under Rockwell-C indentation (HF3), and CC450 samples exhibited moderate delamination (HF5). In contrast, PN400 and CC400 maintained excellent adhesion (HF1–HF2). These findings highlight the importance of balancing hardness improvements with interfacial integrity, suggesting that specific nitriding conditions (e.g., PN450 and CC400) offer the most favorable combination of hardness, corrosion resistance, and adhesion for demanding applications.

Overall, this comparative study demonstrates the versatility of plasma nitriding techniques for AISI 420 martensitic stainless steel and confirms the potential of both PN and CCPN to produce uniform, adherent, and durable nitride layers, making them effective surface engineering strategies for extending the service life of components in aggressive, wear-prone environments.
